# Data-driven development of the nationwide hip fracture registry in the Netherlands

**DOI:** 10.1007/s11657-022-01160-3

**Published:** 2022-12-05

**Authors:** Franka S. Würdemann, Stijn C. Voeten, Janneke A. Wilschut, Inger B. Schipper, Johannes H. Hegeman

**Affiliations:** 1 Department of Traumasurgery, Leiden University Medical Center, Leiden, The Netherlands; 2 Scientific Bureau, Dutch Institute for Clinical Auditing, 2333 AA Leiden, The Netherlands; 3 Department of Traumasurgery, Ziekenhuisgroep Twente, Almelo, Hengelo The Netherlands

**Keywords:** Hip fracture, Registration, Audit, Quality of care, Dataset

## Abstract

***Summary*:**

Additional variables for a nationwide hip fracture registry must be carefully chosen to prevent unnecessary registry load. A registry pilot in seven hospitals resulted in recommending polypharmacy, serum hemoglobin at admittance, and questions screening for risk of delirium to be used in case-mix correction and for development of quality indicators.

**Purpose:**

Clinical registries help improve the quality of care but come at the cost of registration load. Datasets should therefore be as compact as possible; however, variables are usually chosen empirically. This study aims to evaluate potential variables with additional value to improve the nationwide Dutch Hip Fracture Audit (DHFA).

**Methods:**

An expert panel selected eleven new variables for the DHFA, which were tested in a prospective cohort of all hip fracture patients treated in 2018 and 2019 in seven pilot hospitals participating in the DHFA. The association of these eleven variables with complications, mortality, and functional outcomes at 3 months was analyzed using multivariable logistic regression analysis. Based on the results, a proposal for variables to add to the dataset of the DHFA was made.

**Results:**

In 4.904 analyzed patients, three tested variables had significant associations (*p* < 0.01) with outcomes: polypharmacy with complications (aOR 1.34), serum hemoglobin at admittance with complications (aOR 0.63) and mortality (aOR for 30-day mortality 0.78), and a set of questions screening for risk of delirium with complications in general (aOR 1.55), e.g., delirium (aOR 2.98), and decreased functional scores at three months (aOR 1.98).

**Conclusion:**

This study assesses potential new variables for a hip fracture registry. Based on the results of this study, we recommend polypharmacy, serum hemoglobin at admittance, and questions screening for risk of delirium to be used in case-mix correction and for the development of quality indicators. Incorporating these variables in the DHFA dataset may contribute to better and clinically relevant quality indicators.

**Supplementary Information:**

The online version contains supplementary material available at 10.1007/s11657-022-01160-3.

## Introduction

Insight into desired and undesired medical outcomes is of utmost importance for increasing the quality of care. Clinical registries have shown to provide this insight by evaluating and benchmarking quality indicators to improve the quality of care in various health care domains [1, 2]. In clinical registries, patient characteristics and data on organization and outcomes of care are collected in a standardized manner. If the registration load is experienced to be unnecessarily high, this could lead to (selective) missing data with consequences for the analysis of the quality of care [3, 4]. Registry datasets should therefore be as compact as possible, containing only clinically relevant variables and methodologically sound indicators for quality of care.

Registry variables can be divided into patient characteristics, variables describing the organization of care (structure and process), and outcome variables, which provide data on the end result. Quality indicators are mostly composed of process and outcome variables. Structure and process variables usually contain information on care provided by the hospital, and outcome variables generally contain information on recovery, restoration of function, and survival [5]. Sometimes, quality indicators on outcomes are corrected for case-mix using patient characteristic variables. A sound quality indicator is highly distinctive for comparing hospitals and contains a limited set of relevant case-mix variables. Surprisingly, to our knowledge, there is no literature on methodology to select relevant variables for use in registries for clinical auditing [6].

The Dutch Hip Fracture Audit (DHFA), a nationwide hip fracture registry in the Netherlands, was implemented in 2016 [7]. Like other registries, at the start of the registry, variables included in the dataset were chosen by an expert panel based on the national guideline for the treatment of proximal femoral fractures, the guideline for the elderly undergoing surgery, and the Minimum Common Dataset for hip fractures, recommended by the Fragility Fracture Network (FFN MCD) [8–10]. To further improve the quality of the DHFA dataset, the DHFA committee aimed to supplement the dataset with variables of use for case-mix correction of current quality indicators and variables useful for developing new quality indicators. Therefore, this study evaluates potential variables with additional value for the DHFA.

## Methods

### Data collection

Seven hospitals participating in the DHFA prospectively collected an extra set of “test” variables next to the DHFA dataset for this study [7]. All adult hip fracture patients presented in these hospitals and registered in the DHFA between 1–1-2018 and 31–12-2019 were included. Exclusion criteria were periprosthetic fractures and pathological fractures. Data completeness of > 90% for all clinical variables was set as the standard for data quality. Dates of death post-admission were derived from the Dutch Vektis data institute, which collects data from health insurance reimbursements [11]. Using social security numbers, the DHFA and Vektis data are joined by a trusted third party. The researchers were provided with a pseudonymized dataset.

### Variable selection

The selection of variables to be tested for use within the nationwide DHFA was made based upon the literature and after the consensus of the members of an expert panel. This expert panel consisted of six trauma surgeons, one orthopedic surgeon, two geriatricians, one internal medicine specialist, and two clinical researchers. All suggestions for variable selection emanated from the panel discussion during two meetings at the start of the project. Halfway through the inclusion period, an interim discussion was initiated to make adjustments to the chosen variables where necessary. The expert panel agreed to analyze a set of variables for their additional value: five patient characteristics (one of which had several sub-questions), which can be used for case-mix correction of both existing and new quality indicators, and four care process variables that can be used in new quality indicators.

The patient characteristics included were serum hemoglobin level (in mmol/l) at hospital admittance, polypharmacy (use of more than five medications before admission), use of oral anticoagulants*, the Parker Mobility Score at admittance [12] (in order to make a comparison with and validation of the Fracture Mobility score, which is already present in the DHFA dataset), and a set of three screening factors for risk of delirium. The Dutch Healthcare Safety Management System describes the screening tool for elevated risk of delirium in healthcare; the following three questions are asked to the patient or its proxy before admission or within 24 h after admission: (1) Do you have problems with memorization? (2) Did you receive any assistance in any daily living activity in the last 24 h before admission? (3) Are you known with episodes of confusion during earlier sickness or hospitalization [10]?

The last patient characteristic registered is a history of falling in the year prior to fracture, for which the following question was asked to the patient or its proxy: Did you fall once, or more often, in the past 6 months? The registration of this variable was discontinued after the interim panel discussion halfway through the project due to unclear definitions and the processing of the answers to this question.

The four care process variables were administration of tranexamic acids*, the number of consulting medical specialists during admittance (stratified to 1–3 or more than 3), and medical or organizational reason for the delay of surgery (> 48 h after the presentation on the emergency department) and medical or organizational reason for the delay of discharge (> 5 days as this is the median hospital stay in the Netherlands). Medical reasons are all causes of delay that can be attributed to patient-related factors, e.g., the need for preoperative optimization in case of a pre-existent infection or anticoagulant reversal therapy causing a surgical delay. Organizational reasons are all causes of delay related to the organization, e.g., unavailability of an operating theatre or surgical team causing surgical delay or lack of adequate outplacement options causing a delayed discharge.

The interim-expert panel discussion at the end of 2018 led to additional registration of two variables: the use of oral anticoagulants and the administration of tranexamic acid (marked with *). Recording of these variables started on 1–1-2019.

### Outcome parameters

All available outcome measures from the DHFA dataset were divided into three subsets: (1) *in-hospital* outcomes included in-hospital mortality and “any complication in hospital” defined as anemia, delirium, pulmonary embolism, renal dysfunction, pneumonia, urinary tract infections, a fall in hospital, and/or wound infections. Anemia and delirium were also separately analyzed. The exact definitions of complications registered in the DHFA are shown in Supplementary Table 2. (2) *3-month follow-up outcomes* included reoperation (within 16 weeks postoperatively and reported by the hospital), decreased functional mobility (1 or more points higher Fracture Mobility Score at three months when compared to the pre-fracture Fracture Mobility Score [8]), and decreased functional independence (1 or more points higher KATZ-6 ADL score at 3 months when compared to pre-fracture KATZ-6 ADL score [13]). (3) *Post-admission mortality* included 30-day, 90-day, and 1-year mortality. To be included in the analysis of a specific outcome, the patient’s subset data for that outcome needed to be complete.

### Statistical analysis

After 2 years of inclusion, statistical analyses were performed. The between-hospital variation of tested variables was analyzed, as significant differences would emphasize the need for using the variable for case-mix correction in the case of patient characteristics or the need to use the variable as a quality indicator in the case of process or outcome variables. The between-hospital variation was analyzed with descriptive statistics, tested with ANOVA and shown as mean, min, and max % per hospital for each selected variable. The independent associations of each tested variable and the outcomes were analyzed with bivariate and multivariable logistic regression models. For each outcome, tested patient characteristics with a statistically significant bivariate association (*P* < 0.05) were included in a multivariable regression analysis adjusted for possible confounders. Multivariable analysis of tested process variables was impossible due to the circular nature of these variables and tested outcomes.

The included confounders already present in the DHFA were age, sex, fracture side, fracture type, pre-fracture living situation, Fracture Mobility Score [8] and KATZ Index of Independence in Activities of Daily Living (KATZ-6 ADL) score [13], American Society of Anesthesiologist physical status classification (ASA-score) [14], and pre-fracture reported presence of dementia or osteoporosis and risk of malnutrition. The risk of malnutrition was measured during hospital stay using the Short Nutritional Assessment Questionnaire (SNAQ) or the Malnutrition Universal Screening Tool (MUST) and categorized as low (SNAQ 0 or MUST 0), medium (SNAQ 1–2 or MUST 1), or high risk (SNAQ ≥ 3, MUST ≥ 2) [15, 16]. Results are shown as crude odds ratio (OR) or adjusted odds ratio (aOR) with 95% confidence intervals (CI).

To overcome multiple testing, *p*-values < 0.01 were regarded as statistically significant in the multivariable models.

Missing values were analyzed as a separate group in multivariable logistic regression if these exceeded 5% of the total included number of patients. If the missing values in a variable were below 5%, the missing patients were excluded from the analysis. Statistical analysis was performed using RStudio Version 1.1.456 [17].

#### Results

A total of 4904 patients were included, of which 4849 were eligible for analysis of *in-hospital* outcomes, 4421 for *mortality* outcomes, and 2130 for *3-month follow-up outcomes.* Baseline patient characteristics from the DHFA and baseline of the tested variables are shown in Table 1.

**Table 1:**
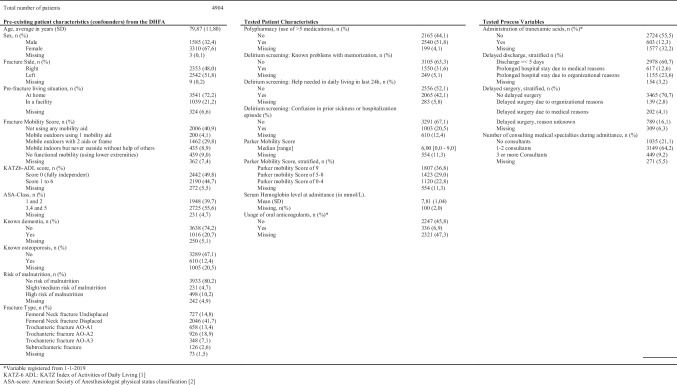
Baseline table of analyzed DHFA variables, tested patient characteristics, and tested process variables

All tested variables had significant between-hospital variation. A summary of mean percentages per hospital is shown in Table 2. The data completeness of the variables “use of oral anticoagulants” and “administration of tranexamic acid” was too low to analyze in regression models. The event rates of the outcomes analyzed are together with the bivariate analysis results, shown in Table 3. Table 4 shows the multivariable analysis for the tested patient characteristics.

**Table 2:** Between hospital variation of potential patient characteristics and process variables

	Mean hospital %	Min hospital %	Max hospital %
Polypharmacy (use of > 5 medications)	52.80	38.50	64.40
Delirium—Known problems with memorization	33.40	26.10	39.20
Delirium—Help needed in daily living in last 24 h	43.20	31.00	53.10
Delirium—Confusion in prior sickness or hospitalization	23.00	13.60	29.60
Parker Mobility score independent inside	54.80	43.60	62.70
Parker Mobility score independent outside	43.30	38.50	48.00
Parker Mobility score independent shopping	42.00	37.00	47.80
Parker Mobility score completely independent	41.90	36.60	47.10
Parker Mobility score completely dependent	1.90	0.70	4.10
Serum Hemoglobin level at admittance (in mmol/L) < 7.5	33.10	29.30	37.60
Oral Anticoagulants	11.60	4.83	28.71
Tranexamic acid	20.00	0.00	40.00
No prolonged hospital stay	63.60	38.70	95.00
Prolonged hospital stay due to medical reasons	12.20	2.30	27.70
Prolonged hospital stay due to organizational reasons	24.00	2.70	48.10
Prolonged hospital stay, reason unknown	0.20	0.00	0.90
No delayed surgery	78.70	0.00	98.60
Delayed surgery due to organisational reasons	3.00	0.00	8.20
Delayed surgery due to medical reasons	4.70	1.10	14.00
Delayed surgery, reason unknown	13.60	0.00	93.20
1–2 consulting physicians	63.60	31.50	85.10
3 or more Consulting physicians	8.60	0.20	21.20

**Table 3:**
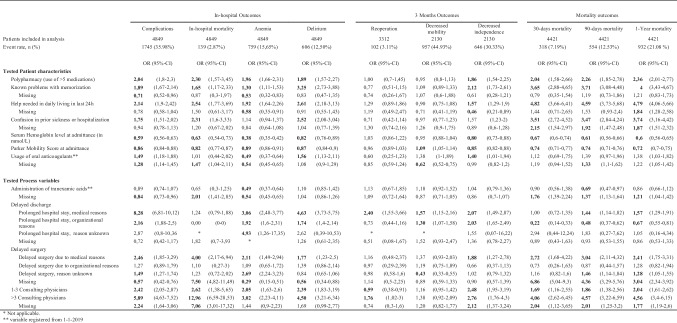
Bivariate analysis of associations between tested patient characteristics and process variables and outcomes

**Table 4:**
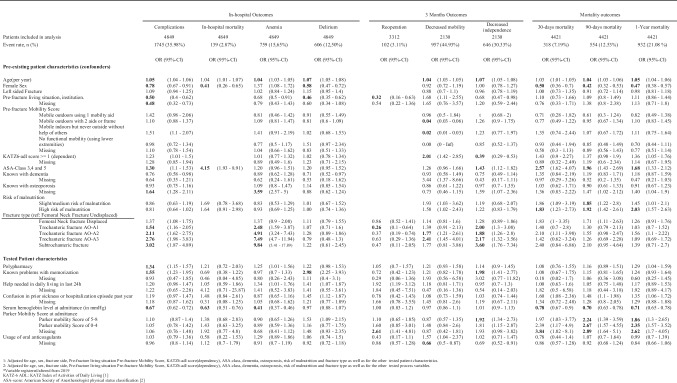
Multivariable analysis of associations between tested patient characteristics and outcomes

### Tested patient characteristics

The multivariable analyses of the association between tested patient characteristics and outcomes are shown in Table 4. Polypharmacy at admittance was registered in 51.8% (Table 1) of the included patients and was associated with in-hospital complications in general (aOR 1.34, *p* < 0.01). Polypharmacy showed no relation with 1-year mortality (aOR 1.29, *p* = 0.02) and anemia (aOR 1.25, *p* = 0.04). The question on memory problems, one out of the three questions to screen for the risk of delirium, was answered positive in 31.6% of the included patients and was associated with the following outcomes: complications in general and delirium in particular, a decrease in independence at three months and also showed bivariate associations with delirium, 30-days, 90 days, and 1-year mortality. The questions “help in daily living” (answered positively in 42.1%, Table 1) and “confusion during prior sickness or hospitalization” (answered positively in 20.5%, Table 1) showed no relation with tested outcomes in the multivariable analysis. The mean serum hemoglobin level (in mmol/l) at admittance was 7.81 (standard deviation of 1.04) and showed statistically significant associations indicating that a lower hemoglobin level is associated with negative outcomes or vice versa—higher levels are protective against negative outcomes. The negative outcomes were complications in general (aOR 0.63, *p* < 0.01), anemia in particular (aOR 0.41, *p* < 0,01), in-hospital mortality (aOR 0.64, *p* < 0.01), 30-day, 90-day, and 1-year mortality (aOR, respectively, 0.78, 0.70, and 0.71 with *p* < 0.01), Higher Parker Mobility Scores at admittance of 5–8 that were associated with decreased independence at three months (aOR 1.92, *p* < 0.01), and 90-day and 1-year mortality (aOR 2.24 and 1.86, respectively, with *p* < 0.01).

### Tested process variables

The results of the bivariate analysis of associations between tested process variables and outcomes are shown in Table 3. 60.7% of the included patients were discharged within 5 days. The most common reasons for the delayed discharge were organizational reasons (23.6%, Table 1). The bivariate analysis showed delayed discharge for organizational reasons to be statistically significantly associated with in-hospital complications (OR 2.16, *p* < 0.01) and decreased mobility (OR 1.30, *p* < 001), and independence at 3 months (OR 2.03, *p* < 0.01). Delayed discharge for medical reasons was associated with decreased mobility (OR 1.57, *p* < 0.01) and independence (OR 2.07, *p* < 0.01) at 3 months post-fracture and with increased 90-day (OR 1.44, *p* < 0.01) and 1-year mortality (OR 1.57, *p* < 0.01). 70.7% of the included patients were operated on within 48 h after the presentation in the emergency ward (Table 1). Delayed surgery due to organizational reasons showed no associations with tested outcomes. Delayed surgery due to medical reasons was associated with decreased independence at 3 months (OR 1.88, *p* < 0.01) and mortality at 30 and 90 days and 1 year (OR 2.72, OR 3.04, and OR 2.41 with *p* < 0.01, respectively). Having been seen by more than three consulting medical specialties was associated with increased in-hospital mortality (OR 12.96, *p* < 0.01).

## Discussion

This study analyzed variables with potential for case-mix correction or the development of new quality indicators for the Dutch Hip Fracture Audit. Several tested variables showed associations with outcomes. Polypharmacy was associated with complications, and screening for delirium was associated with complications (e.g., delirium) and decreased independence after 3 months. Serum hemoglobin levels showed a reversed continuous association with mortality (in hospital and up to 1 year) and complications in hospital. Higher Parker Mobility Scores were associated with decreased independence at 3-month follow-up and with mortality up to 1 year. Other relevant findings were the associations between the process variable “delayed discharge due to organizational reasons” and complications and decreased functional outcomes and the association between the process variable “number of consulting specialists” and in-hospital mortality.

Polypharmacy was associated with complications and mortality. Polypharmacy is often named in the context of multimorbidity and frailty, which are known to be a predisposition for adverse outcomes after an event like a fractured hip [18, 19]. Registration of comorbidities would probably depict a more accurate pre-fracture state of the patient, but this comes with extensive registry loads. Therefore, polypharmacy can be a proxy for multiple morbidities and can be used in case-mix correction for quality indicators for complications and mortality.

In the current literature, the incidence of delirium in hip fracture patients reaches to 25% [20–22]. Delirium accelerates cognitive decline and worsens outcomes, while it is partly preventable [22–24]. Surprisingly, only a few national registries use quality indicators for the occurrence and prevention of delirium. Moreover, the few existing indicators on delirium do not seem to use case-mix correction [6]. Registration of the screening questions for delirium can be useful in two ways, as it showed associations with mortality and delirium. It can be used for case-mix correction in mortality quality indicators and a quality indicator on (prevention of) delirium.

There were significant associations between serum hemoglobin levels at admittance and complications in general, specifically anemia and mortality at several time points. The association with serum hemoglobin levels at admittance and the registration of the complication anemia is explainable: the definition of anemia in the DHFA includes the indication for a blood transfusion (Supplementary Table 2). The chance of needing a blood transfusion is higher a priori when a patient has lower serum hemoglobin at admittance. Whether the patient surely received a blood transfusion is not registered within the DHFA. The found association with mortality is of interest. The relation between serum hemoglobin levels at admittance and mortality has been thoroughly examined. Most studies use cut-off values for lower hemoglobin levels, so results are not easily comparable with the continuous value we have tested. However, like our results, studies suggest that lower serum hemoglobin levels are associated with excess mortality [25]. Measurement of serum hemoglobin is usually standard of care at admission; it is relatively inexpensive and an objective parameter [25]. The serum hemoglobin level at admittance can be used as a case-mix factor for mortality outcomes, and further research on the effect of this parameter on complications is indicated.

Higher Parker Mobility Scores were associated with decreased independence at 3-month follow-up and with mortality up to 1 year. The DHFA already registers mobility using the Fracture Mobility Score. In our study, the pre-fracture Fracture Mobility Scores did not show significant associations with decreased mobility, whereas the Parker Mobility Score did. Therefore, replacing the Fracture Mobility Score might seem indicated. However, several reasons are found to opt for maintaining the Fracture Mobility Score. The Fracture Mobility Score has been recently validated to the Parker Mobility Score and is deemed valid to measure the mobility of hip fracture patients [12]. Besides that, it has a lower registration load, is easier to measure because it has fewer and simpler questions, and the Fragility Fracture Network advises its use [8, 12].

The analysis of the tested process variables was hindered by the lack of information on the temporal sequence of events. For this reason, we refrained from multivariable analyses. However, for some parameters, the bivariate analysis results could lead to improvement of care. Firstly, the process variable delayed discharge due to organizational reasons was associated with complications. This could mean patients who stay in the hospital longer than their medical situation requires are prone to suffer complications. Consequently, in-hospital complications have severe consequences for hip fracture patients. They are related to decreased functional outcomes and quality of life, higher mortality rates, and increased health care costs [26, 27]. However, studies on preventing complications in hip fracture patients usually do not examine organizational factors other than the involvement of a geriatrician [28, 29]. Evidence on the effect of complications on length of stay in hip fracture patients is widely available. However, studies on this association in a reversed sequence, extended lengths of stay leading to complications, are lacking [30–32]. These findings underline the importance of dealing with organizational issues within the hospital. They indicate the need for future research on the main organizational problems causing a delay of discharge in this specific patient category and how to improve this.

The second process variable showing an association of interest is the number of consulting medical specialists, which, in case this number was high, was associated with higher in-hospital mortality. One should notice this is an uncorrected bivariate analysis; therefore, these patients likely have a fragile pre-fracture health state suffering multiple comorbidities explaining part of the association. Besides this, definitions could have been formulated more strictly, e.g., did the involvement of a geriatric specialty count as a consulting specialty, or is this incorporated in the standard of care? Therefore, the definition of this variable should be improved. Nonetheless, in this study with an explorative nature, the high OR for mortality should be pointed out. One can hypothesize that many consulting medical specialists, especially if no specific one is in the lead, could cause unclarity or even interference in care policies, which might lead to suboptimal care for the patient. Therefore, we believe this parameter to be of interest for future studies.

Comparing the DHFA dataset with eight datasets of other national hip fracture registries, we have found that none of these was registering polypharmacy at admittance [33–40]. Several other registries included screening for delirium at admittance, although they used different risk scores [33, 38, 40]. Serum hemoglobin at admittance is only recorded within the German national hip fracture registry amongst other laboratory results [38]. It is unclear for what use these laboratory results are registered. Regarding which potential case-mix factors are included in other registries, all used ASA scores and pre-fracture functioning and mobility scores [33–40]. Polypharmacy, the serum hemoglobin at admittance, and screening for risk of delirium could be valuable to improve their case-mix models. Regarding the delayed surgery and discharge, we have found that all registries recorded discharge dates. However, none was registered whether there was a delay and when patients were medically cleared for discharge, nor did they include information on the number or type of consulted medical specialists. We suggest further research before adding these to datasets for the latter process variables.

The suggested new variables are shown in Box 1, and the complete dataset for the Dutch Hip Fracture Audit, including suggested variables, is shown in Supplementary Table 1.

Box 1 Advised variables as additions for the DHFA dataset.

**Table:** 

Variable	Type	Class	Option set	Use
Polypharmacy at admittance (> 5 medications)	Patient	Factor	0: No, 1: Yes, 9: Unknown	Case-mix for: Complications Mortality
Combined: Delirium screening: Decreased memory Delirium screening: Help in daily living Delirium screening: Confusion in prior sickness/hospitalization	Patient Patient Patient	Factor	0: No, 1: Yes, 9: Unknown	Case-mix for: Complications Anemia specifically Delirium specifically Functional outcomes at 3 months Mortality
Serum hemoglobin at admittance in mmol/L	Patient	Integer (one decimal)	NA	Case-mix for: Complications Anemia specifically Mortality

### Strengths and limitations

This study included a substantial number of patients. The data used is of high quality: post-admission decease dates were derived from a trustable data source, and the participating hospitals validated reoperation data. The method described in this study is innovative; to the authors’ knowledge, no research on a structural approach to identifying variables with potential for registries has been published.

These analyses have several limitations. The first and foremost limitation is how the process variables were defined and registered. Due to the lack of information on the temporal sequence of events, we needed to refrain from multivariable analyses. Another limitation is the amount of missing data, especially on outcomes. However, for the clinical variables, > 90% completeness was achieved. Due to the large number of tests, a *p*-value of < 0.01 was chosen. Still, multiple testing may have led to unjustly significant findings.

### Future perspectives

An important lesson was learned in this study: we have to clearly define variables for a clinical registry and ensure they have enough distinctive power to address questions or knowledge gaps adequately. To exemplify, the benefits of gaining insights into reasons for postponed surgery and delayed discharge could be substantial, as they may form a leading point in reducing health care costs. However, registering this information does not suffice its goal without timelines on events such as when a specific complication occurred or for which exact reason discharge was delayed. This should always be considered when considering adding parameters to a registry.

The data of hip fracture databases can be used to develop an Extended Common Dataset for hip fractures, in which essential information for case-mix correction is incorporated. Several research questions and quality domains in hip fracture care can be further explored with these new hip fracture registry variables. This study aimed to gain insight into the relevance of each potential parameter. For this purpose, simplified outcome measures are used, e.g., decreased mobility or independence, and future perspectives include in-depth analyses using, e.g., exact functional scores or specific reoperations and complications.

For registrations in general, lowering registration loads and meanwhile developing new quality indicators is not the easiest combination. This study may spark the interest of other registries and set a standard to analyze variables before adding them to their registry datasets.

## Conclusion

In this study, we have structurally assessed variables of potential additional value for hip fracture registries. The assessment led to the recommendation to add polypharmacy, serum hemoglobin at admittance, and three screening questions on the risk of delirium to the Dutch Hip Fracture Audit Dataset. These variables have proven statistical associations with outcomes. Incorporating these variables may lead to better and clinically more relevant quality indicators for hip fracture care in the near future.

### Supplementary Information

Below is the link to the electronic supplementary material.

Supplementary file1 (PDF 213 KB) 

## Data Availability

Data and coding are available on request; please get in touch with the corresponding author.
