# Supplementary material for: Data-driven development of the nationwide hip fracture registry in the Netherlands
Source: Arch Osteoporos. 2022 Dec 5;18(1):2. doi: 10.1007/s11657-022-01160-3 (PMC9719884; doi:10.1007/s11657-022-01160-3)
Supplement: Supplementary file 1 — Supplementary file1 (PDF 213 KB) [file 11657_2022_1160_MOESM1_ESM.pdf]

Supplemental Table 1. Current - and suggested additional- parameters of the Dutch Hip Fracture Audit Dataset

Variable	Variable type	Format	Options
Id	Patient	Integer, 9 numbers	
Country	Patient	Factor	Option set with all countries of the world
Social service number	Patient	Integer, 9 numbers	
Initials	Patient	Text	
Name	Patient	Text	
Surname	Patient	Text	
Name of Partner	Patient	Text	
Postal code	Patient	String	
Date of birth	Patient	dd-mm-yyyy	
Sex	Patient	Factor	1: Male, 2: Female, 7: Undefined
Decease date (if applicable)	Patient	dd-mm-yyyy	
**CLINICAL SECTION**			
Date and time of arrival in Emergency Room	Process	dd-mm-yyyy hh:mm	
Specialty of practitioner in charge	Process	Factor	5: Traumasurgeon, 6: Ortho-traumasurgeon, 7: surgeon, 8: Ortho-surgeon, 9: Geriatrician, 10: Internal medicine-elderly, 11: Internal medicine, 12: Nursing home physician
Consulting specialty	Process	Factor	5: Traumasurgeon, 6: Ortho-traumasurgeon, 7: surgeon, 8: Ortho-surgeon, 9: Geriatrician, 10: Internal medicine-elderly, 11: Internal medicine, 12: Nursing home physician
Transferred patient	Process	Factor	0: No, 1: Yes, 9: Unknown
Type of geriatric involvement	Process	Factor	0: None, 1: Post-operative consult, 2: Peri-operative shared, 3: Shared geratric-traumatology ward
Date and time of departure Emergency Room	Process	dd-mm-yyyy hh:mm	
Pre-fracture living setting	Patient	Factor	1: Home, 2: Home with help in daily living, 3: Nursing home, 4: Revalidation, 7: other
Pre-fracture Fracture Mobility Score	Patient	Integer	1 to 5
Pre-fracture KATZ6-ADL dependency score	Patient	Integer	0 to 6
Known with dementia	Patient	Factor	0: No, 1: Yes, 9: Unknown
Known wit osteoporosis	Patient	Factor	0: No, 1: Yes, 9: Unknown
SNAQ or MUST-score for malnutrition	Patient	Integer	
Fracture side	Patient	Factor	1: Right, 2: left, 3: bilateral
Fracture type	Patient	Factor	1: Femoral neck undisplaced, 2: Femoral neck displaced, 3: Trochanteric AO-A1, 4: Trochanteric AO-A2, 5: Trochanteric AO-A3, 6: Subtrochanteric, 9: Unspecified
ASA-class	Patient	Factor	1 to 5
Type of treatment	Process	Factor	1: Conservative, 2: Hemiarthroplasty, 3: Cannulated screws, 4: Total hip arthroplasty, 5: Sliding hip screw, 6: Intramedullary nailing, 10: Girdle stone.
Date and time of start of Operation	Process	dd-mm-yyy hh:mm	
Augmentation used	Process	Factor	0: No, 1: Yes
Anesthesia complete	Process	Factor	0: No, 1: Yes
Anesthesia regional	Process	Factor	0: No, 1: Yes
Anesthesia spinal	Process	Factor	0: No, 1: Yes
Complication in general	Outcome	Factor	0: No, 1: Yes
Specific complication: anemia	Outcome	Factor	0: No, 1: Yes
Specific complication: cardiac decompensation	Outcome	Factor	0: No, 1: Yes
Specific complication: pressure ulcers	Outcome	Factor	0: No, 1: Yes
Specific complication: delirium	Outcome	Factor	0: No, 1: Yes
Specific complication: pulmonary embolism	Outcome	Factor	0: No, 1: Yes
Specific complication: renal failure/insufficiency	Outcome	Factor	0: No, 1: Yes
Specific complication: pneumonia	Outcome	Factor	0: No, 1: Yes
Specific complication: urinary tract infection	Outcome	Factor	0: No, 1: Yes
Specific complication: fall during admission	Outcome	Factor	0: No, 1: Yes
Specific complication: wound infection	Outcome	Factor	0: No, 1: Yes
In-hospital mortality	Outcome	Factor	0: No, 1: Yes
Date of discharge	Process	dd-mm-yyyy	
Osteoporosis screening planned at moment of discharge	Process	Factor	0: No, 1: Yes
Discharge destination	Process		
		Factor	1: Home, 2: Home with help in daily living, 3: Nursing home, 4: Revalidation, 7: Other
**FOLLOW-UP SECTION**			
Date of follow-up information gained	Process	dd-mm-yyyy	
Deceased < 3 months	Outcome	Factor	0: No, 1: Yes, 9: Unknown
Reoperation	Outcome	Factor	0: No, 1: Yes, 9: Unknown
Reason for reoperation (if applicable)	Outcome	Factor	1: Infection, 2: Dislocated device, 3: Peri-prosthetic fracture, 4: Hematoma, 5: Loosening of device, 6: Luxation of endoprothesis, 7: Operative treatment after conservative, 9: Unknown
Date of reoperation (if applicable)	Outcome	dd-mm-yyyy	
Living situation at three months	Outcome	Factor	1: Home, 2: Home with help in daily living, 3: Nursing home, 4: Revalidation, 7: Other
Mobility score at three months	Outcome	Integer	1 to 5
KATZ6-ADL dependency score at three months	Outcome	Integer	0 to 6
**Suggested additions after methodological testing and expert panel discussion:**			
Serum Hemoglobin at admittance (in mmol/L)	Patient	Integer (one decimal)	
Polypharmacy at admittance (use of >5 medications)	Patient	Factor	0: No, 1: Yes, 9: Unknown
Delirium screening: Known problems with memorization	Patient	Factor	0: No, 1: Yes, 9: Unknown
Delirium screening: Help needed in daily living in last 24h	Patient	Factor	0: No, 1: Yes, 9: Unknown
Delirium screening: Confusion in prior sickness or hospitalization	Patient	Factor	0: No, 1: Yes, 9: Unknown

KATZ-6 ADL: KATZ Index of Activities of Daily Living [1]
ASA-score: American Society of Anesthesiologist physical status classification [2]
SNAQ: Short Nutritional Assessment Questionnaire [3]
MUST: Malnutrition Universal Screening Tool [4]

Supplemental Table 2. Definitions used to register complications in the Dutch Hip Fracture Audit Dataset

Variable	Definition used
Anemia	Indication for blood tranfusion according to the 4-5-6 rule deducted from the Dutch Transfusion Guideline, 2011:<br>Hb <4 mmol/L:<br>- Acute blood loss in ASA-I patients, <60 years old, normovolemic, blood loss at 1 locus.<br>Hb <5 mmol/L:<br>- Acute blood loss in ASA-I patients, >60 years old, normovolemic, blood loss at 1 locus<br>- Acute blood loss in ASA-I patients, <60 years old, normovolemic, blood loss at >1 locus. (polytrauma patients)<br>- Patient <60 years old, preoperatively in case of an expected blood loss >500 mL<br>- Fever<br>- Postoperatively after uncomplicated open cardiac surgery<br>- ASA II en ASA III<br>Hb <6 mmol/L:<br>- ASA-IV patients<br>- Patients not able to increase the cardiac minute volume in compensation of hemodilution.<br>- Septic/toxic patient.<br>- Patients with severe pulmonary disease.<br>- Patients with symptomatic cerebrocardiovascular disease.
Cardiac decompensation	Indication to start diuretics AND<br>Clinical presentation of cardiac decompensation (dyspnea, tachypnoea, pulmonary crepitations, elevated central venous pressure, edema, enlarged liver, enlarged ictus, cardiac murmur, 3rd heart sound AND/OR<br>Elevated proBNP or signs of pulmonary congestion on x-thorax.
Pressure ulcers	Open skin, pressure sores from grade 2-4, definition by Shea et al. [5]
Delirium	Diagnosed in concordance with the Diagnostic and Statistical Manual of Mental Disorders (DSM-5) criteria and noted in the clinical record. [6]
Pulmonary embolism	Diagnosed pulmonary embolism as described in the guideline 'Antitrombotic therapy' [7]
Renal failure/insufficiency	Decrease in renal function (Increase in serum creatine ≥ 26,5 uml/l in 48h or increase of ≥1.5 times the baseline concentration in 7 days)
Pneumonia	Clinical presentation of pneumonia, for which antibiotic therapy is initiated.
Urinary tract infection	Clinical presentation of urinary tract infarction, for which antibiotic therapy is initiated.
Fall during admission	Unintentionally coming to the ground or some lower level, definition by the Kellogg International Work Group [8]
Wound infection	Clinically presentation of wound infection for which antibiotic therapy is initiated.

References

1. Katz S, Ford AB, Moskowitz RW, et al (1963) Studies of Illness in the Aged: The Index of ADL: A Standardized Measure of Biological and Psychosocial Function. JAMA J Am Med Assoc 185:914–919. https://doi.org/10.1001/jama.1963.03060120024016
2. ASA Physical Status Classification System | American Society of Anesthesiologists (ASA). https://www.asahq.org/standards-and-guidelines/asa-physical-status-classification-system. Accessed 25 Jan 2021
3. Kruizenga HM, Seidell JC, de Vet HCW, et al (2005) Development and validation of a hospital screening tool for malnutrition: The short nutritional assessment questionnaire (SNAQ©). Clin Nutr 24:75–82. https://doi.org/10.1016/j.clnu.2004.07.015
4. Elia M (2001) The Malnutrition Advisory Group consensus guidelines for the detection and management of malnutrition in the community. Nutr Bull 26:81–83. https://doi.org/10.1046/j.1467-3010.2001.00111.x
5. Shea JD (1975) Pressure sores: classification and management. Clin Orthop Relat Res 89–100
6. APA (2013) American Psychiatric Association, 2013. Diagnostic and statistical manual of mental disorders (5th ed.)
7. Startpagina - Antitrombotisch beleid - Richtlijn - Richtlijnendatabase. https://richtlijnendatabase.nl/richtlijn/antitrombotisch_beleid/antitrombotisch_beleid_-_korte_beschrijving.html. Accessed 13 Apr 2022
8. Kellogg International Work Group (1987) The prevention of falls in later life. A report of the Kellogg International Work Group on the Prevention of Falls by the Elderly. Dan Med Bull 34 Suppl 4:1–24
